# Early and Late Induction of *KRAS* and *HRAS* Proto-Oncogenes by Reactive Oxygen Species in Primary Astrocytes

**DOI:** 10.3390/antiox6030048

**Published:** 2017-06-29

**Authors:** Samantha Messina, Erika Di Zazzo, Bruno Moncharmont

**Affiliations:** 1 Department of Human Sciences, Society and Health, University of Cassino and Southern Lazio, Cassino 03043, Italy; 2 Department of Medicine and Health Sciences “Vincenzo Tiberio”, University of Molise, Campobasso 86100, Italy; erika.dizazzo@unimol.it (E.D.Z.); moncharmont@unimol.it (B.M.)

**Keywords:** p21^Ras^, *KRAS*, *HRAS*, astrocytes, reactive oxygen species

## Abstract

Astrocytes, one of the predominant types of glial cells, function as both supportive and metabolic cells for the brain. Among mammalian tissues, the highest levels of p21^Ras^ protein are detected in the brain. Here, we investigated the expression of *KRAS* and *HRAS* proto-oncogenes in primary astrocytes following acute oxidative stimulation. Reactive oxygen species (ROS) changed the expression of proto-oncogenes at both transcriptional and translational levels. De novo protein synthesis analysis measured approximate values of proteins half-life, ranging from 1–4 h, of the different H- and K- isoforms by western blot analysis. Quantitative gene expression analysis of *KRAS* and *HRAS* revealed an unexpected short-term induction of *KRAS* mRNA in primary astrocytes in response to acute stimulation. Indeed, cultured astrocytes responded to proteasomal inhibition by preventing the reduction of c-K-Ras. A fraction of K-Ras protein accumulated in the presence of ROS and cycloheximide, while a substantial proportion was continuously synthesized. These data indicate that ROS regulate in a complementary fashion p21^Ras^ isoforms in primary astrocytes: K-Ras is rapidly and transiently induced by post-translational and post-transcriptional mechanisms, while H-Ras is stably induced by mRNA accumulation. We suggest that K-Ras and H-Ras are ROS sensors that adapt cells to metabolic needs and oxidative stress.

## 1. Introduction

The small guanine nucleotide binding proteins of the p21^Ras^ family, encompassing in mammals the highly homologous H-Ras, N-Ras, and K-Ras isoforms, are actively expressed in mouse astrocytes [1,2,3,4]. Each of the three mammalian *RAS* genes encodes a membrane-associated 21-kDa protein that acts as a molecular switch to convey extracellular-derived signals into the cell interior. p21^Ras^ proteins cycle between an inactive Guanosine Diphosphate (GDP)-bound state and an active Guanosine-5′-triphosphate (GTP)-bound state, because of the concerted action of Guanine nucleotide Exchange Factors (GEFs) and GTP-ase Activating Proteins (GAPs) [5]. The H- and K-Ras are targets of reactive oxygen species (ROS) [6,7] and also regulate in a reciprocal fashion the redox state of cells by inducing Mn-superoxide dismutase (SOD) (K-) [8], or Nicotinamide Adenine Dinucleotide Phosphate (NADPH) oxidase complex via Extracellular signal–Regulated Kinases (ERK) (H-) [9,10,11]. Expression and/or activation of H-Ras is regulated by ROS in fibroblasts [12] and Jurkat cells [7]. Induction of *HRAS*, *NRAS*, and *KRAS* endogenous mRNA has been reported in cultured mouse astrocytes by pro-inflammatory cytokine Interferon-gamma (INF-γ) treatment [2].

The best-characterized p21^Ras^ effectors are the Raf kinases, through which p21^Ras^ activates the Mitogen-Activated Protein Kinase (MAPK) cascade, the Phosphatidylinositol-4,5-bisphosphate 3-kinase (PI3Ks), and a family of Ral Guanine nucleotide exchange factors (GEFs). Although p21^Ras^ proteins are ubiquitously expressed, differences in their expression in various tissues and during development suggest that each isoform may exert a specialized and specific function. However, most of the studies on p21^Ras^ signaling are based on enforced expression of p21^Ras^ mutant protein [8,9,10,11,12], which, because of its mechanism of action cannot discriminate between contributions from different isoforms or from other members of the p21^Ras^ superfamily. This limitation can be partly overcome by analyzing wild-type individual isoforms endogenously expressed at physiological expression levels [3,4,13,14,15].

Oxidative stress has been implicated in aging and many human diseases, notably neurodegenerative disorders. In particular, oxidative stress in the Central Nervous System (CNS) occurs after ischemic, traumatic, or excitotoxic insults, when the excessive generation of ROS overwhelms the intracellular antioxidant capacity [16]. Neurons are highly sensitive to oxidative damage, whereas astrocytes exert a protective function acting as cell scavengers and producing neurotrophic factors in response to ROS [17,18,19,20,21,22]. Astrocytes respond to ROS by activating MAP kinases (MAPKs), including the extracellular signal-regulated kinases ERK1/2, JUN kinases (JNKs), and p38 MAPKs [23,24,25]. The monomeric GTP-binding protein, p21^Ras^, has an established role in activating these pathways and has been implicated in the cellular response to oxidative stress [24,25] and is a common signaling target of reactive free radicals and cellular redox stress [7]. However, how individual p21^Ras^ proto-oncogenes *KRAS* and *HRAS* react to oxidative stimuli is still poorly understood. This manuscript describes the expression of wild-type K-Ras and H-Ras in primary astrocytes following acute oxidative stimulation, with the reactive oxygen species playing a key role. ROS appears to affect the expression of isoforms (a) in discrete ways and (b) at both the transcriptional and translation levels.

## 2. Experimental Procedures

*Reagents—*Monoclonal antibody (Ab-3) anti-pan Ras, proteasome inhibitor MG132 (carbo-benzoxy-L-leucyl-L-leucyl-L-leucinal), and MEK-inhibitor PD98059, cycloheximide (CHX) Transforming Growth Factor beta (TGF-β), and Bone Morphogenetic Protein 4 (BMP4) were purchased from Calbiochem (San Diego, CA, USA). The antibodies against ERK1/2, phospho-ERK1/2 and H-Ras (sc-520), K-Ras (sc-521), and N-Ras (F155) and antibody-conjugated beads H259 anti-pan Ras were purchased from Santa Cruz Biotechnology (Santa Cruz, CA, USA). The anti-β-actin antibody, hydrogen peroxide solution, MTT (3-(4,5-dimethylthiazol-2-yl)-2,5-diphenyl tetrazolium bromide) were purchased from Sigma-Aldrich Ltd. (St. Louis, MO, USA); the monoclonal anti-caspase-9 precursor and its cleaved form were from Upstate (Waltham, MA, USA). Dnase I and Improm II RT were purchased by Promega (Madison, WI, USA). The Horseradish Peroxidase (HRP)-conjugated secondary antibodies (mouse and rabbit) and the Enhanced Chemiluminescence (ECL) detection system were from Amersham-Pharmacia (Piscataway, NJ, USA). Fetal Calf Serum (FCS) and Horse Serum, Trypsin- Ethylenediaminetetraacetic acid (EDTA), and Penicillin/Streptomycin solutions were purchased from HyClone Europe Ltd. (South Logan, UT, USA); Modified Eagle’s Medium (MEM) and Dulbecco Modified Eagle’s Medium (DMEM) (GIBCO BRL, Life Technologies, Rockville, MD, USA), Trizol reagent, and Lipofectin reagent were from Life Technology (Carlsbad, CA, USA). SYBR^®^Green and 2X Supermix cocktail were from Bio-Rad (Hercules, CA, USA).

*Cell Culture Conditions—*Primary astrocytes from the cerebral cortices of neonatal CD1 mice (Charles River Laboratories International, Inc., Wilmington, MA, USA) were cultured as described previously [3,4]. Confirmation of astrocyte-enriched cultures were assayed by immuno-staining utilizing anti- Anti-Glial Fibrillary Acidic Protein (GFAP) antibody (data not shown). All experiments were carried out in confluent cultures of primary mouse astrocytes, grown in complete (20% serum-containing) medium for 15–20 days in vitro (DIV). We then replaced the medium with fresh medium containing 20% FCS or 2% FCS and then added H_2_O_2_ at indicated concentrations. The treatments of cultured astrocytes were as follows: pre-treated or not with the proteasome-inhibitor MG132 40 μM for 15–20 min in complete medium; pre-treated or not with CHX 20 μM for 15–20 min in complete medium; pre-treated or not with the MEK-inhibitor PD98059 40 μM for 15–20 min in complete medium and re-administered concomitantly with hydrogen peroxide for the indicated times. Human Embryonic Kidney (HEK-293) cells were purchased from American Type Culture Collection, ATCC (Cat. No. CRC-1573) (ATCC, Manassas, VA, USA). Transient transfection of Ras recombinant construct (constitutively active K-Ras carrying a Val-12 mutation or a double mutant H-Ras carrying Leu-61 and Ser-186 mutations), to assess the specificity of K-Ras and H-Ras antibodies, was performed in HEK-293 cells following the standard procedures.

*MTT-Viability Assay—*To measure the cytotoxic potential of the treatment, an MTT (3-(4,5-dimethylthiazol-2-yl)-2,5-diphenyl tetrazolium bromide) assay was carried out as described before [26]. Briefly, mouse astrocytes cells were prepared as described above and plated on uncoated 24-well primary culture plates and grown for 15–20 days. Thereafter, the growth medium was removed and fresh medium (500 μL/well) was added and cells were treated with the indicated hydrogen peroxide concentration for the indicated times. Ten microliters of MTT solution (5 mg/mL in Phosphate-Buffered Saline (PBS) was added to the wells containing 100 mL medium, and the plates were incubated for 4 h in humidified 95% air and 5% CO_2_ atmosphere at 37 °C. Thereafter, the medium was removed and 100 mL of solubilization solution (isopropyl alcohol/0.04 M HCl) was added and incubated for a few min to dissolve the water-insoluble formazan salt. Quantification was then carried out with an ELISA reader at 595 nm, using a 655 nm filter as reference. Data are expressed as percentage of the untreated controls, and values represent the means ± SD of four wells each of three independent experiments.

*Western Blot Analysis—*Cultured astrocytes or HEK-293 cells were washed with PBS and lysed for 15 min in ice-cold Radioimmunoprecipitation (RIPA) buffer (1% Triton X-100, 0.5% deoxycholate -DOC), 0.1% Sodium Dodecyl Sulphate (SDS), 50 mM Tris pH 7.6, 150 mM NaCl, 1 mM Phenyl-Methyl-Sulfonyl Fluoride (PMSF), 1 mg/mL aprotinin, leupeptin and pepstatin and, in the proteasomal experiments (MG132), supplemented with 2 mM N-ethylmaleimide (NEM). Cell lysates were clarified at 13,000 rpm for 30 min at 4 °C and the cytosolic fraction was immediately subjected to protein determination using a Bradford colorimetric assay (Bio-Rad Laboratories Inc., Hercules, CA, USA) or subjected to immunoprecipitation procedures.

*Immunoprecipitation Procedures—*The cytosolic fraction from treated astrocytes was processed for immunoprecipitation with anti-pan Ras conjugated beads (H259). One milligram of lysate was incubated with 4 μg of antibody-conjugated beads for 2 h at 4 °C. Then the beads were subjected to an extensive wash with RIPA buffer and subjected to western blot analysis following the standard procedures after Sodium Dodecyl Sulphate-PolyAcrylamide Gel Electrophoresis (SDS-PAGE) electrophoresis on polyacrylamide gels (7–15%). Densitometric analysis of western blot data was carried out using a computer-based microdensitometer (NIH Image Software, Bethesda, MD, USA).

*Oxidative Species Detection—*2,7-dichlorofluorescein diacetate (H_2_DCF-DA, Sigma-Aldrich Co., St. Louis, MO, USA) was used to measure oxidative species by flow cytometry. Non-fluorescent H_2_DCF-DA was cleaved by endogenous esterases and then was oxidized to generate fluorescent DCF. Confluent cortical mouse astrocytes were maintained 15–20 days in vitro (DIV) in complete medium, and then the growth medium was switched into a serum-free medium containing or not hydrogen peroxide for varying time periods. Cells were incubated with 10 μM H_2_DCF-DA for 1 h, harvested by mechanical scraping, washed twice with cold PBS, resuspended in 500 μL of PBS, and analyzed by flow cytometry FACScan Flow Cytometer (Becton Dickinson, Franklin Lakes, NJ, USA). Experiments were performed in triplicate. The data were acquired and analyzed by CELLQUEST software (Becton Dickinson, Franklin Lakes, NJ, USA).

*Activity Ras Assay*—p21^Ras^ activity was assayed using glutathione S-transferase (GST)-Ras binding domain (RDB) fusion protein, corresponding to the human RBD, residues 1-149 of Raf-1, expressed in *Escherichia coli (E. coli)*, bound to glutathione agarose [27]. Active p21^Ras^ proteins were pulled-down following the protocol described. Briefly, cells were lysed in Mg^2+^ lysis buffer (MLB) containing 25 mM (4-(2-hydroxyethyl)-1-piperazineethanesulfonic acid (HEPES), pH 7.5, 150 mM NaCl, 1% Igepal CA-630, 10 mM MgCl_2_, 1 mM EDTA, and 2% glycerol. Ten micrograms of Raf-1 RBD agarose was added to 1 mg of cell lysates and samples were gently rocked at 4 °C for 30 min. The agarose beads were collected by centrifugation at 1600× *g* for 10 min at 4 °C and washed three times with MLB buffer. The amount of active p21^Ras^ was assessed by western blot using antibodies specific against isoforms. Pull-down assays on recombinant isoforms were performed with 200–500 μg of total lysates from HEK-293 cells transiently transfected with p21^Ras^ mutant plasmids.

*Quantitative polymerase chain reaction (qRT-PCR Assay)—*Total RNA was extracted from the cultures and subjected to DNA-se I treatment according to the manufacturer’s instructions. One microgram of total RNA was then employed for cDNA synthesis using Improm II RT and random hexamer primers according to the manufacturer’s instructions. The RT product was diluted to 100 μL with sterile, distilled water and 1 μL of cDNA was subsequently used as template for 25 μL of PCR containing iNOS, *KRAS* and *HRAS* primers. The following primers were used: *KRAS* forward TTGCCTTCTAGAACAGTAGACA; reverse TTACACACTTTGTCTTTGACTTC; β-actin: forward, TGAACCCTAAGGCCAACCGTG; reverse, GCTCATAGCTCTTCTCCAGGG; inducible nitric oxide synthase (*iNOS*) forward, GGTGTTCTTTGCTTCCATGCTAAT, reverse, GTCCCTGGCTAGTGCTTCAG; *HRAS* forward GTGACCTGGCTGGTCGCACTG; reverse CACTTGCAGCTCATGCAGCC. Real-Time Quantitative SYBR^®^ Green conditions included an initial denaturation step of 95 °C for 5 min followed by 40 cycles of 95 °C for 30 s, 55 °C for 1 min, and 72 °C for 30 s. Standards, samples, and negative controls (no template) were analyzed in triplicate. Concentrations of mRNA were calculated from serially diluted standard curves simultaneously amplified and normalized with respect to the recombinant construct wild-type K-Ras.

*Ethics Statement—*All primary cultures used in this study were obtained from cerebral cortices of CD1 neonatal mice (Charles River Laboratories, Calco (Lecco) Italy) as previously described [4]. Cells for primary culture were obtained from newborn mice after decapitation under ether anesthesia, and every effort was made to minimize suffering. All experiments were carried out according to the European (86/609/EEC) and Italian (D: Lgs. 116/92) guidelines of animal care. 

*Statistical Analysis*—Unless otherwise stated, all experiments were performed at least three independent times. All data are presented as the means ± S.D. of at least three experiments in triplicates (*n* ≥ 9). Statistical significance between groups was determined using Student’s *t*-test (matched pairs test or unmatched test were used as indicated in the figure legends). All statistical analyses were performed using JMP Software purchased by Statistical Discovery SAS Institute (*p* < 0.05, statistical significance; *p* < 0.001, high statistical significance).

## 3. Results

**Figure 1:**
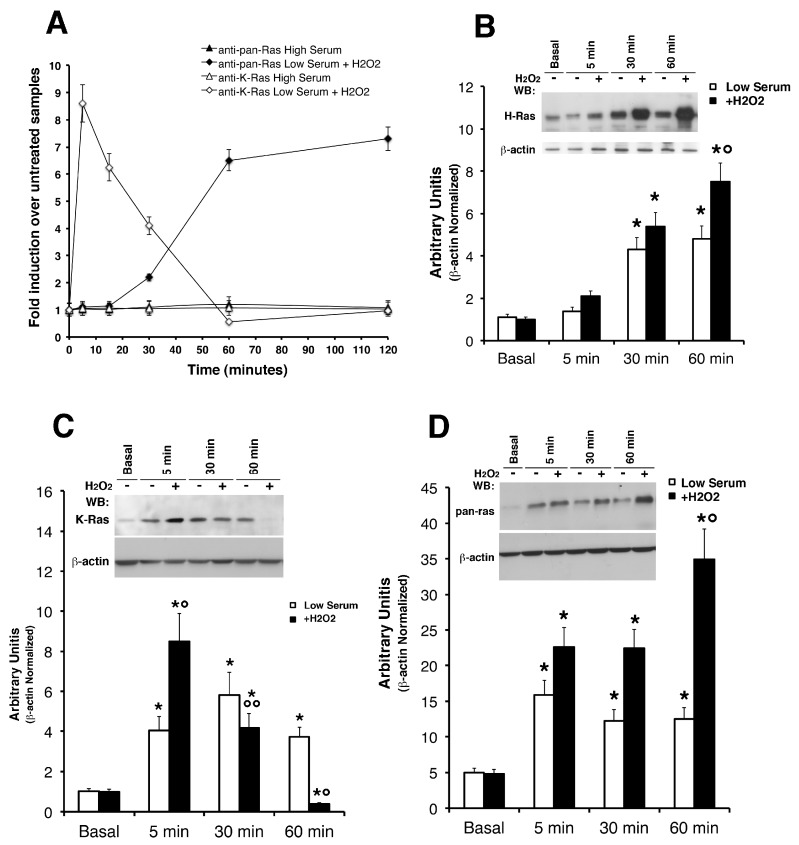
Reactive oxygen species (ROS) selectively induce K-Ras and H-Ras protein levels in primary astrocytes. The direct effects of hydrogen peroxide addition in regulating the protein levels of K-Ras and H-Ras in cultured astrocytes were measured by western blot analysis. The kinetics of total p21^Ras^ expression and K-Ras isoform in cultured astrocytes subjected to H_2_O_2_ treatment is shown in (**Panel A**). The growth medium (high-serum 20%) was switched into a low-serum (2%) medium containing or not H_2_O_2_ and western blot analyses were performed on Radioimmunoprecipitation (RIPA) buffer extracts. Total p21^Ras^ protein shows constitutive low levels in untreated astrocytes and increases upon oxidative stimulation (**Panel D**). K-Ras (sc-521) levels increased in 5 min and rapidly decreased, reaching the basal levels at 60 min of stimulation (**Panel C**). Conversely, H-Ras protein levels peaked at 60 min and remained high for up to 120 min (**Panel B**); representative immunoblots with antibodies against H-Ras (sc-520) and pan-Ras (H259) show the same immune-reactivity pattern with differences in sensitivity. Monoclonal anti-β actin was used as loading control. Experiments were carried out in triplicates and statistical significance obtained by Student’s *t*-test. * *p* < 0.01 as compared with the untreated normal astrocytes (Student’s unpaired test); ° *p* < 0.02 as compared with the untreated sample (Student’s matched pairs test).

*Reactive Oxygen Species (ROS) Selectively Induce K-Ras and H-Ras Protein Levels in Primary Astrocytes—*We studied the direct effects of hydrogen peroxide addition in regulating the protein levels of K-Ras and H-Ras in cultured astrocytes. Firstly, we tested the effects of increasing concentrations of H_2_O_2_ on survival of primary astrocytes by MTT assay (Supplementary Figure S1). The figure shows treatment with physiologically relevant H_2_O_2_ doses, similar or less than those recorded post-ischemia [28], does not exert a cytotoxic effect on cultured astrocytes. We see no evidence of H_2_O_2_ toxicity in astrocytes up to 500 μM, as shown in Supplementary Figure S1 (panel A and C) in complete medium (20% serum) or in low serum (2%) in which this effect is weakened (panel B). The insensitivity of astrocytes in this range of concentrations may be ascribed to their high hydrogen peroxidase activity and their ability to clear H_2_O_2_ from culture medium [29,30]. Our experiments show that mild oxidative insults, as subtoxic H_2_O_2_, strongly activate astrocytic Ras pathways by acute stimulation. Conversely, mouse astrocytes subjected to lethal H_2_O_2_ exposure 1–2 mM show Caspase-9 activation of the apoptosis-mediated pathway, as shown in panel D (Supplementary Figure S1D). We next asked if the concentration of H_2_O_2_ utilized in this study does not measurably alter cellular ROS levels. 2,7-dichlorofluorescein diacetate (H_2_DCF-DA) was used to measure oxidative species by flow cytometry (Supplementary Figure S2). Cultured astrocytes were pre-loaded with 10 µM 2,7-dichlorofluorescein diacetate (H_2_DCF-DA) in complete medium for 30 min, and then subjected to H_2_O_2_ in increasing doses. Representative FACS data of DCF mean intensity show a shift to the right (i.e., increase of ROS) in treated samples, as expected, thus validating our readout system. All subsequent experiments were done with H_2_O_2_. Next, we stimulated the cells with 500 µM H_2_O_2_ and measured the levels of K-Ras and H-Ras isoforms by western blot (Figure 1) with specific antibodies against K-, H-, and total p21^Ras^ (see antibody specificity assayed in Supplementary Figure S2). Figure 1 shows that K-Ras levels, in cells exposed to H_2_O_2_ in low serum, increased in 5 min and rapidly decreased, reaching the basal levels at 60 min of stimulation (panel C). Under the same conditions, phosphorylation of ERK1/2 were induced by H_2_O_2_ with the same kinetics (Supplementary Figure S2 panel C). Conversely, the pan- Ras and hence H-Ras peaked at 60 min (panel B and D). It is worth noting that, N-Ras isoform was not recognized (probably less immunogenic) in all the western blot experiments on mouse astrocytes. We performed experiments comparing (on the same SDS-PAGE) mouse astrocytes with human glioblastoma tissues, as shown in Supplementary Figure S3, with a strong and sustained N-Ras signal detected in cancer samples with the same antibody (see Supplementary Figure S4 and Methods). Next, we attempted to address an important point, namely the effects of ROS on Ras activity. To correlate p21^Ras^ levels with its activity, we measured the binding capacity of Ras to the binding domain of Raf (Supplementary Figure S4). The GTP-bound form of p21^Ras^ was pulled-down from total fresh lysates of cultured astrocytes using the glutathione S-transferase (GST)-Ras binding domain (RDB) fusion protein containing residues 1–149 of Raf-1. Both isoforms showed slight activation (panel B) compared to recombinant plasmid overexpressed in HEK-293 cells (panel A). The data suggest that ROS transiently elevate K-Ras activity in the short term but do not alter H-Ras activity. Considered jointly, these data show differences in protein levels with differential kinetics: K-Ras is rapidly induced and downregulated in 60 min, H-Ras is induced at 60 min and remains high for up to two hours (Figure 1 panel A). Indeed, as TGF-β1 increases ROS production and suppresses antioxidant enzymes, leading to a redox imbalance, we have performed additional experiments using this cytokine as an alternative to ROS sources. Moreover, we have previously shown that trophic deprivation induces an intracellular burst of ROS in cultured astrocytes [4]. We used TGF β1 and BMP4 accordingly with previous reports on optic head astrocytes [28,29]. Supplementary Figure S5 shows western blot analysis on mouse astrocytes treated with BMP4 and TGFß. It is worth noting that we have not used oxygen-glucose deprivation (OGD), an in vitro ischemia model, because it induces oxidative stress in neurons but not in astrocytes [31].

**Figure 2:**
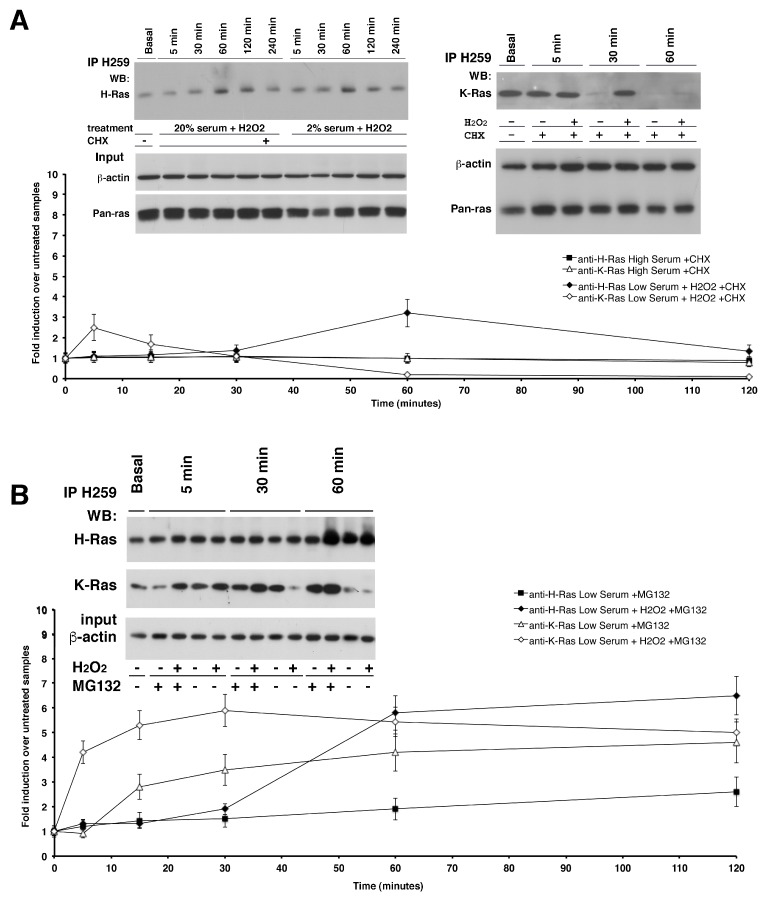
Post-transcriptional regulation of K-Ras and H-Ras proteins by H_2_O_2_-induced oxidative stress. Western Blot analysis was performed on Y13-259 from astrocytes RIPA extracts that were cycloheximide (CHX) pre-treated or proteasomal inhibitor MG132 pre-treated and quickly subjected to H_2_O_2_ exogenous administration in complete medium or low-serum medium. In (**Panel A**), cultured astrocytes were pre-treated with the drug (CHX, 20 µg/mL) and the decay of the target protein over time was determined by western blot analysis with specific antibodies against H-Ras and K-Ras (**left and right panel A, respectively**). The addition of H_2_O_2_ shortened the expression of H-Ras (t_1/2_ values 1 h); conversely, H_2_O_2_ was able to induce K-Ras protein levels even in the absence of mRNA translation. Western blot analysis of total Ras (pan-Ras Ab-3) and monoclonal β-actin was performed in the same experiment and used as loading control (bottom panel). (**Panel B**) shows immunoblot analysis of H- and K- isoforms from immunoprecipitates of astrocytes pre-treated with the proteasome inhibitor, MG132, in the presence or absence of H_2_O_2_. Proteasomal inhibition prevented the reduction of K-Ras at 30 min, which maintained at high levels for up to 60 min. K-Ras protein also increased in cells treated with MG132, without H_2_O_2_, with kinetics mirroring mRNA accumulation. Total kinetics of K- and H- protein levels are shown in the bottom graph. All data derive from three independent experiments performed in triplicate (mean ± SD; *n* = 9).

*Post-Transcriptional Regulation of K-Ras Protein Levels by ROS—*To study the mechanism of ROS induction of K-Ras levels, we inhibited protein translation with cycloheximide (CHX) and stimulated the cells with H_2_O_2_. Figure 2 shows an immunoprecipitation assay (see Experimental Procedures) with antibody-conjugated beads Y13-H259 anti-pan Ras from one milligram of astrocytes total lysate treated or not with subtoxic doses of H_2_O_2_. Western blot analysis with specific antibodies against the two isoforms revealed that ROS were able to induce K-Ras protein levels even in the absence of mRNA translation in both serum conditions (Figure 2A, right panel). However, comparing K-Ras protein levels in cells exposed or not to cycloheximide in the presence of ROS (Figure 1 and Figure 2), we noted that upon ROS stimulation a significant fraction of protein (60–70%) was synthesized de novo, because it was not detectable when translation was inhibited by cycloheximide. Collectively, these data suggest that 30–35% of K-Ras protein levels are controlled at post-transcriptional level and 70% are induced by mRNA accumulation. To investigate the mechanism of post-translational regulation of K-Ras levels, we treated the cells with the proteasome inhibitor, MG132, in the presence of H_2_O_2_. Figure 2B shows that in the presence of MG132, the levels of K-Ras did not fall as in control cells at 30 min but remained high for up to 120 min. K-Ras protein increased also in cells treated with MG132, without H_2_O_2_, with a kinetics-mirroring RNA accumulation, although not at levels seen in cells stimulated with H_2_O_2_ (Figure 2B). This result suggests that the newly synthesized protein is subjected to rapid degradation. In the same cells and under the same conditions, H-Ras protein levels did not change (Figure 2B). These data indicate that a fraction of K-Ras protein levels is subjected to proteasomal degradation. Indeed, we assayed the inhibition of vesicle recycling by treatment with the Na(+)/H(+) ionophore Monensin, observing that it doesn’t change K-Ras protein levels neither the compound PD98059, a non-ATP competitive MEK inhibitor (Supplementary Figure S6A,B).

**Figure 3:**
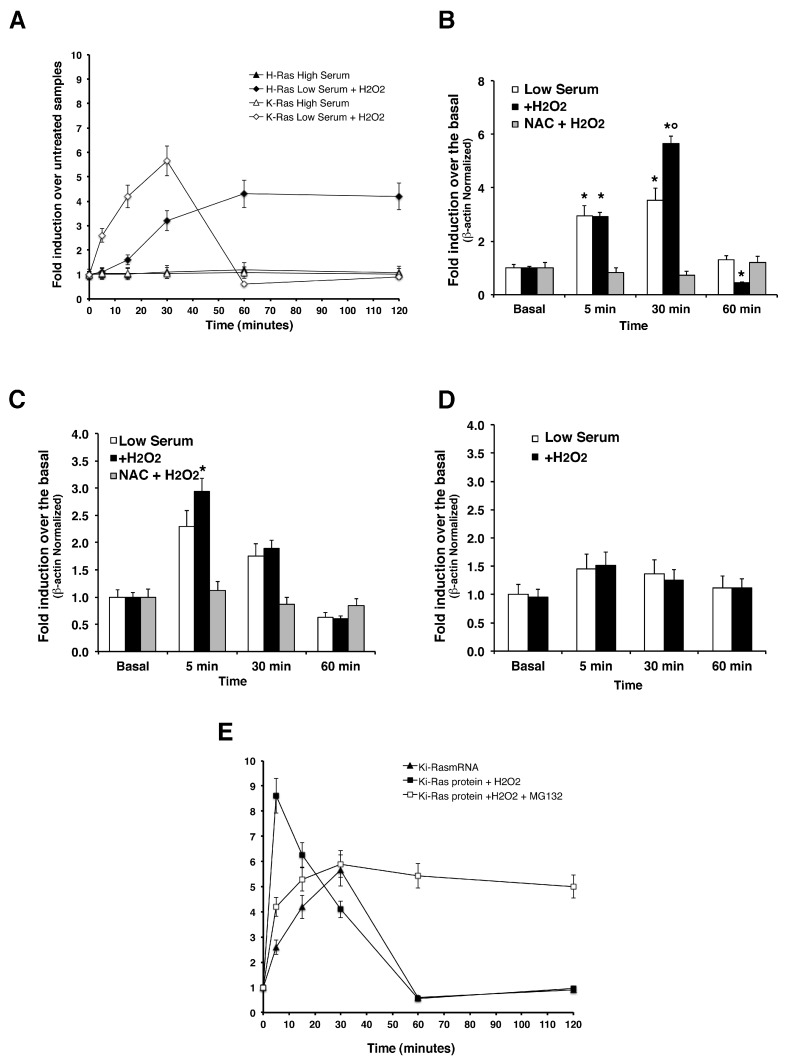
Proto-oncogene K-Ras is transcriptionally induced by H_2_O_2_-induced oxidative stress in primary astrocytes. Quantitative polymerase chain reaction (qRT-PCR) analysis of *KRAS*, *HRAS*, and *iNOS* in cultured astrocytes subjected to H_2_O_2_-induced oxidative stress or untreated (low-serum lacking H_2_O_2_ exogenous administration). The **Panel A** graph summarizes differences in total kinetics of K-Ras and H-Ras mRNA induction by H_2_O_2_ treatment in cultured astrocytes. **Panel B**, **C**, and **D** show mRNA fold induction of K-Ras, iNOS, and H-Ras, respectively, in primary astrocytes in presence or absence of N-Acetyl-cysteine (NAC) and in presence or absence of H_2_O_2_. To examine whether Reactive Oxygen Species (ROS) formation was causally related to the mRNA increase, cultures were pre-treated with 5 mM N-acetylcysteine for 5 h prior to the medium switch. This pre-treatment dampened the mRNA induction in both conditions. **Panel E** summarizes translational and transcriptional K-Ras kinetics in astrocytes exposed to ROS. All experiments were carried out in triplicates and statistical significance obtained by one-way ANOVA analysis with Dunn’s post hoc * *p* < 0.01 and ° *p* < 0.05.

*Transcriptional Induction of HRAS* and *KRAS —*Since cycloheximide experiments indicate that a fraction (60 to 70%) of K-Ras protein is controlled at transcriptional or post-transcriptional levels, we measured *KRAS* and *HRAS* mRNA levels by quantitative PCR. Figure 3 shows that *KRAS* was induced by H_2_O_2_ at 30 min. Under the same conditions, H-Ras protein and mRNA levels increased synchronously upon H_2_O_2_ treatment and reached a plateau at 60 min of ROS challenge. Indeed, we measured *iNOS* mRNA levels in the same experiments and Figure 3C shows a sharp induction of inducible NOS enzyme in mouse astrocytes, which is in line with previous results by [25] showing the importance of p21^Ras^ signaling in regulating the inducible nitric oxide synthase (*iNOS*) in primary astrocytes. Accordingly, it has been shown that Ras modification has a role in the amplification of *iNOS* levels [32,33]. These data indicate that ROS induce steady H- mRNA levels in primary astrocytes. *KRAS* mRNA levels in cells exposed to ROS accumulate steadily at 30 min and decay at 60 min. There is a clear discrepancy between K-Ras protein and mRNA levels: K-Ras protein rises early relative to mRNA (5 min versus 30 min) and decays at 30 min. In comparing K-Ras mRNA and protein peaks in cells exposed to ROS and in the presence of MG132, we conclude that the early increase of the protein at 5 min is essentially post-translational (see Figure 3E). At later times (30–60 min), RNA decays, but the protein can be maintained at high levels by inhibiting the proteasome. In conclusion, these data suggest that ROS rapidly stimulate a fraction of K-Ras protein by increasing mRNA levels at 5–30 min. Another fraction of the protein (approximately 30–35% of the total) instead, is degraded by the proteasome, also in the absence of active translation. The signaling downstream of Ras, such as ERK1/2 does not influence K-Ras protein levels after ROS induction. In fact, inhibition of ERK1/2 in the presence of ROS did not affect K-Ras levels in ROS-exposed cells (Supplementary Figure S4C).

## 4. Discussion

In our present study, we clearly demonstrated that reactive oxygen species (ROS) regulate K-Ras and H-Ras proto-oncogenes with different transcriptional, translational, and post-translational mechanisms in primary astrocytes. The mRNA transcript of *KRAS* proto-oncogene shows low-abundance levels, compared to the heavily transcribed *HRAS*, in primary astrocytes and is induced by INF-γ treatment [2]. We report that *KRAS* mRNA is rapidly induced by oxidative stimuli, and this is consistent with data from gene expression profiling that reveals *KRAS2* involvement in the upregulation of hypoxia-responsive genes in primary human astrocytes [33]. Our data show that protein stability and rate of translation are both modulated by ROS within a definite time window. While, mutational activated mammalian K-Ras is considered to be a long-lived protein [34], the endogenous p21^Ras^ half-life ranges from six to nine hours in non-transformed cells [35]. Our data demonstrate that wild-type Ras proteins possesses a very short half-life (owing to constitutive rapid proteasomal degradation) and K-Ras isoform levels are regulated via two temporally distinct mechanisms. In the early phase, K-Ras partially accumulates in the absence of translation (fraction 30–35%) (Figure 2A) and, in the late phase, is stabilized by MG132 only in the presence of ROS (Figure 2B). Of note, proteasome activity may be impaired through the irreversible oxidation of functionally important cysteine residues as a consequence of oxidative stress [36]. Our hypothesis is that some of the cysteine within 19S subunit of the proteasome are targets for modification by ROS. Further support for this conjecture was provided by a study by Nabhan et al. that showed that the substitution of Cys-120 substantially decreased the activity of 19S subunit towards some substrates [37]. The remaining fraction (70%) is inhibited by cycloheximide and stabilized by MG132 and only slightly modified by ERK1/2 (Supplementary Figure S3). These data strongly suggest that K-Ras may be localized in two subcellular compartments: one containing the synthesized protein (old), which is mobilized by ROS; the other, the newly synthesized protein, which seems to be continuously degraded by the proteasome. Remarkably, de novo synthesized K-Ras was reported to ‘exit’ the sequence of post-translational modifications on the surface of the Endoplasmic Reticulum (ER) at an unknown stage and reach the plasma membrane through an anomalous route [38]. Of note, ubiquitylation of wild-type K-Ras presumably is due to differential subcellular localization or binding partners [39]. Analysis of H-Ras levels indicates that ROS induce protein and its mRNA with the same kinetics, suggesting that the two Ras isoforms complement each other in cells exposed to ROS—K-Ras is acutely but transiently induced, while H-Ras is, instead, chronically stimulated. These data seem to contradict previous observations made in primary human fibroblasts exposed to Platelet-derived Growth Factor (PDGF), in which H-Ras was induced by ROS with a short half-life (15–30 min), whereas K-Ras was rather stable (two hours) [12]. The apparent contradiction is resolved because the total GTP-binding activity and the total p21^Ras^ levels are comparable in the two systems. In primary astrocytes, H-Ras is stable, K-Ras instead is rather unstable after ROS stimulation. H-Ras and K-Ras control ROS production in opposing fashion: H-Ras stimulates NADPH-oxidase (NOX) enzymes [11], while K-Ras stimulates mitochondrial SOD [10]. The final product(s) of H- and K-Ras downstream signaling is/are very similar in fibroblasts and astrocytes, although these two isoforms are regulated in a reciprocal fashion. This is further suggested by the selective action of ERK1/2 on H- and K-Ras levels in fibroblasts and astrocytes. H-Ras levels are regulated by ERK1/2 and ROS, while K-Ras levels are regulated only by ROS [12] (Figure 1, Supplementary Figure S2 and S3). Hyper-activated K-Ras is associated with overproduction of ROS, as reported in a cancer context [40], and, moreover, astrocytes gliosis is associated with hyper-activation of Ras signaling pathways induced by oncogenic K-Ras [41]; it is conceivable that astrocytic Ras-dependent pathways are important mediators of neuroprotective adaptive responses to oxidative stress. Although further investigations are clearly needed, considered jointly, these observations indicate that the loop linking ROS to Ras isoforms protein levels is common to mammalian non-malignant cells and may provide a selective and differential response to oxidative stress in terms of time and space.

## Supplementary Materials

The following are available online at www.mdpi.com/2076-3921/6/3/48/s1, **Figure S1.** MTT Cell viability assay. **Figure S2.** Oxidative species detection. **Figure S3.** N-Ras is almost undetectable in primary astrocytes. **Figure S4.** Detection of Ras activation by GST-Raf-RBD. **Figure S5.** TGFβ treatment induces H-Ras and K-Ras in cultured astrocytes. **Figure S6.** H- and K-Ras protein levels in cells exposed to H_2_O_2_ and treated with Monensin and MEK inhibitor, PD98059.
