# Supplementary material for: Early and Late Induction of KRAS and HRAS Proto-Oncogenes by Reactive Oxygen Species in Primary Astrocytes
Source: Antioxidants (Basel). 2017 Jun 29;6(3):48. doi: 10.3390/antiox6030048 (PMC5618076; doi:10.3390/antiox6030048)
Supplement: Supplementary file 1 [file antioxidants-06-00048-s001.pdf]

antioxidants

MDPI

Supplementary Materials

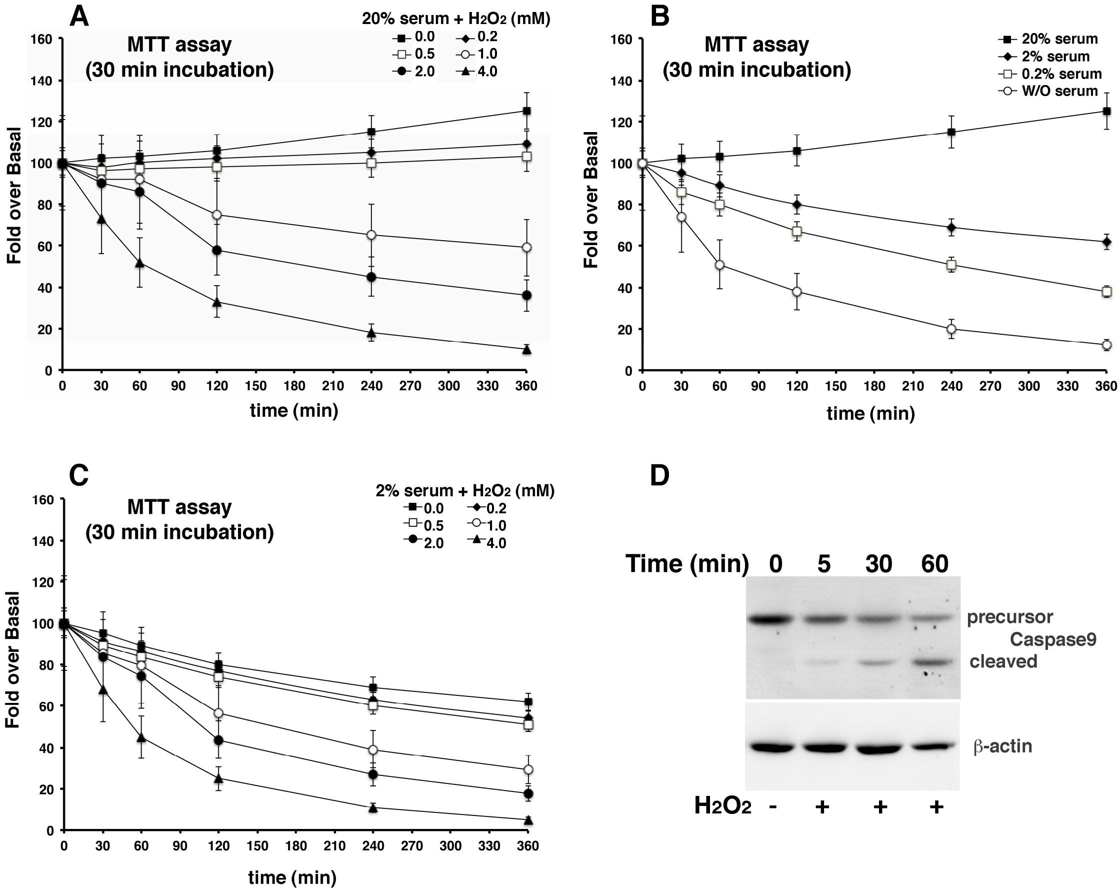

Figure S1. MTT Cell viability assay. To measure the cytotoxic potential of the oxidative treatment, the MTT [3-(4,5-dimethylthiazol- 2-yl)-2,5-diphenyl tetrazolium bromide] assay was carried out as reported in Experimental Procedures. We also checked for signalling cascade activated downstream by lethal H_2O_2-induced oxidative stress. **Panel A and C** show proliferation curves in high-serum and low-serum condition with increasing doses of H_2O_2 ranging from subtoxic to lethal doses (0.2 mM to 4.0 mM). **Panel B** reports survival rates of proliferation with different low- to high- serum containing medium. In **Panel D** is shown a representative western blot assay for cleaved caspase-9 in cultured astrocytes subjected to lethal oxidative stress (2mM). RIPA extracts from cultured astrocytes exposed for indicated times to H_2O_2 shows protein levels of cleaved caspase-9 were significantly increased after 30 and 60 min resulting in extrinsic cell death pathways. Values are means ± SEM of 4–6 determinations. *$p < 0.05$ (Student's *t*-test) versus the respective controls (absence of treatment).

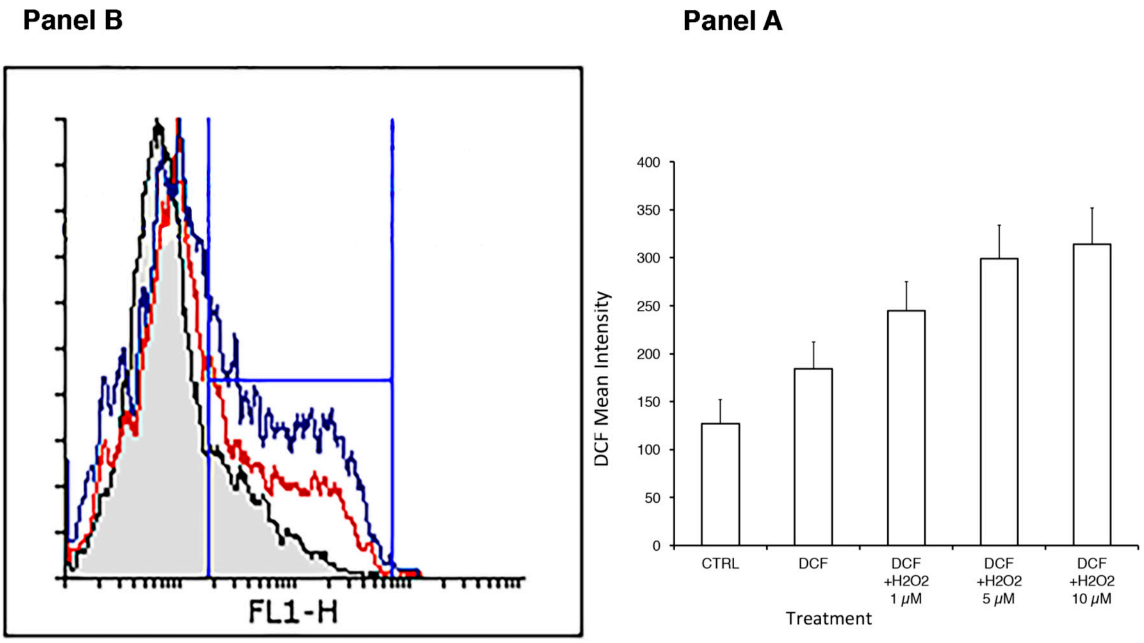

Figure S2. Oxidative species detection. Fluorescent- activated cell sorting (FACS) analysis of intracellular reactive oxygen species (ROS) formation is shown in Panel A. Cultured astrocytes were pre-loaded with 10 µM 2,7-dichlorofluorescein diacetate (H2DCF-DA) in complete medium for 30 min, and then subjected to H_2O_2 increasing doses. In order to set up the system, gating of live cells and staining of unstressed cells with H2DCF-DA was performed. **Panel A** shows representative FACS data overlay of band of cells without fluorescence probe (grey), representing background, and cells stained with H2DCF-DA (FL-l) in the presence of H_2O_2 (red and blue). In order to assess endogenous reactive oxygen species H2DCF-DA was used and the intensity of the resulting fluorescence was measured by FACS as described. **Panel B** shows quantitative measurement of the number of cells (counts) in the *y*-axis (DCF mean intensity) measured at indicated samples treatment vs untreated in the *x*-axis. Experiments were performed in triplicate. The data were acquired and analyzed by CELLQUEST software (Becton Dickinson).

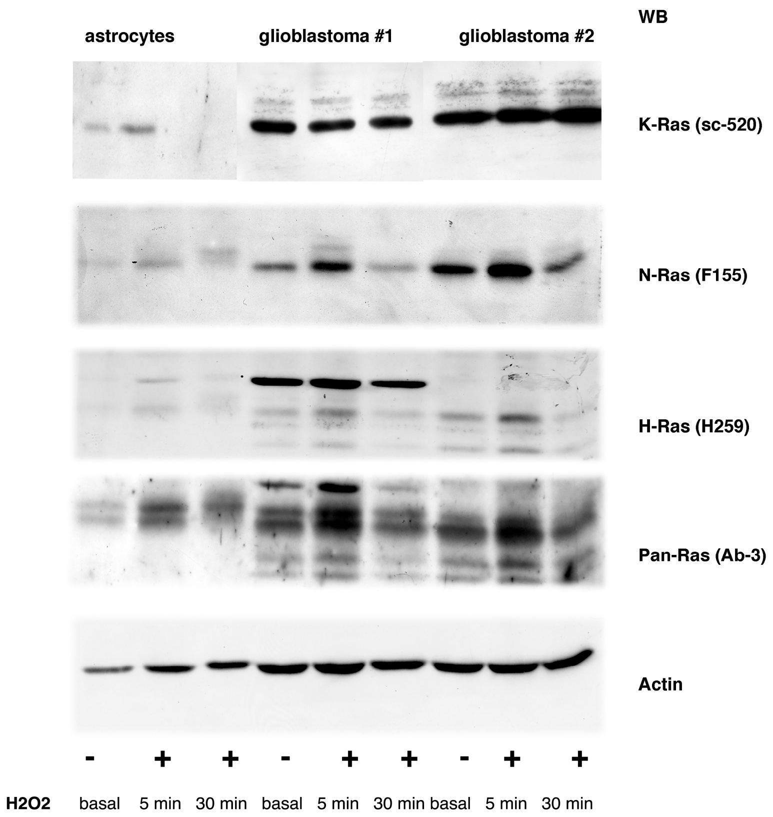

Figure S3. N-Ras is almost undetectable in primary astrocytes. Figure shows Western Blot analysis of H-, N- and K-Ras protein levels in astrocytes compared glioblastomas exposed to H_2O_2. Primary astrocytes and glioblastomas were exposed for the indicated times to 500 μM H_2O_2 for the indicated times. Total extracts were prepared and analyzed by immunoblot with specific antibodies to H-Ras (H259), N-Ras (F155), K-Ras (sc-520) and Pan-Ras (Ab-3). Western blot analysis of monoclonal β actin was performed in the same experiment and used as loading control.

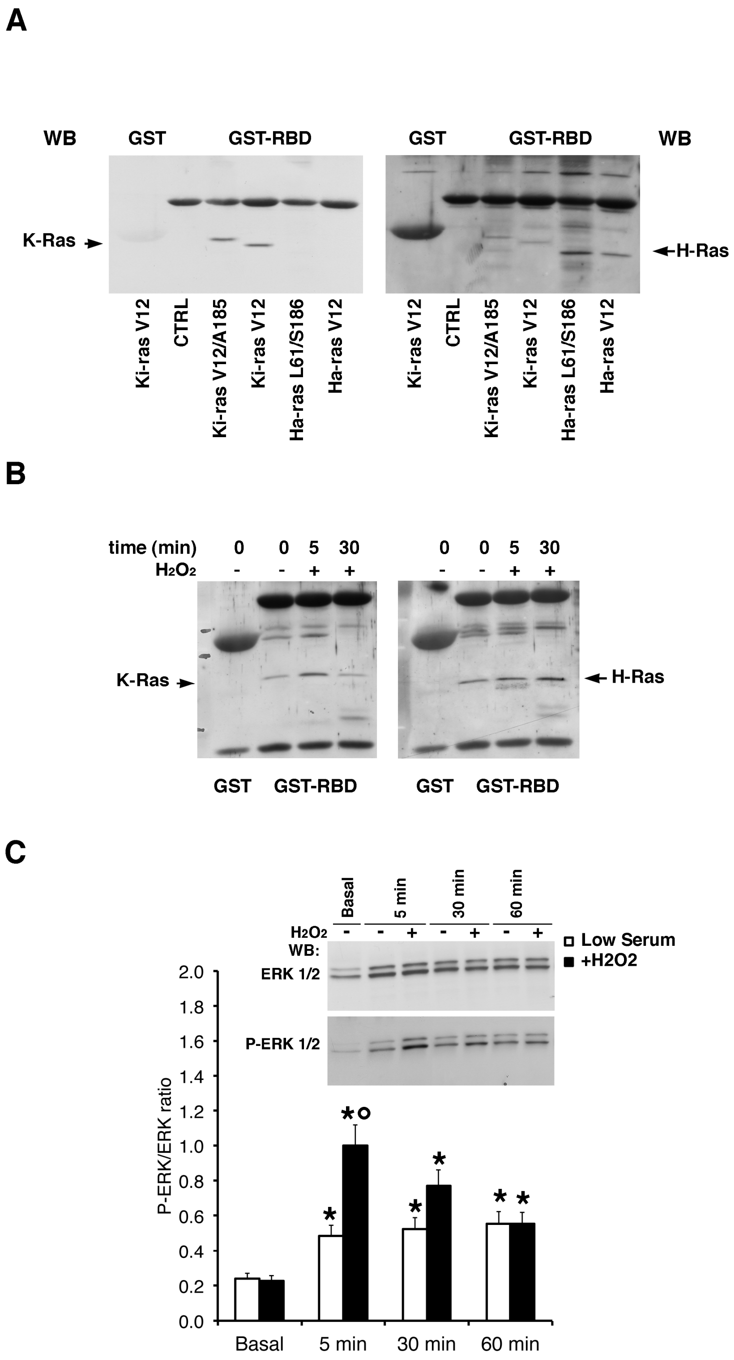

Figure S4. Detection of Ras activation by GST-Raf-RBD. Active Ras was measured by its ability to bind GST-Raf-RBD in control HEK-293 cells compared with primary astrocytes treated or not with H_2O_2. Antibody specificity recognizing H-Ras (sc-520) and K-Ras (sc-521) was assessed by Western Blot on pulled-down samples. **Panel A shows** HEK-293 cells transfected with the activated version of H-Ras-Val 12 and K-Ras-Val 12 plasmids and subjected pull-down assay with GST-Raf-RBD 48 h later (see Experimental Procedures). Conversely, **Panel B** shows immunoblot analysis on pulled-down samples from primary astrocytes challenged with H_2O_2 for indicated times. In **Panel C** is shown the MAPK pathway activation downstream redox stimulation. Western Blot analysis of total and phosphorylated form of ERK1/2 kinase in primary astrocytes. Cultured astrocytes were exposed 5, 30 and 60 min to 500 μM H_2O_2 in low serum in presence or absence H_2O_2. Semi-quantitative analysis of protein phosphorylation is reported in below graph. All experiments were carried out in triplicates and statistical significance obtained by Student's t-test. *P<0.01 as compared with the untreated normal astrocytes (Student's unpaired test); °P<0.02 as compared with the untreated sample (Student's matched pairs test).

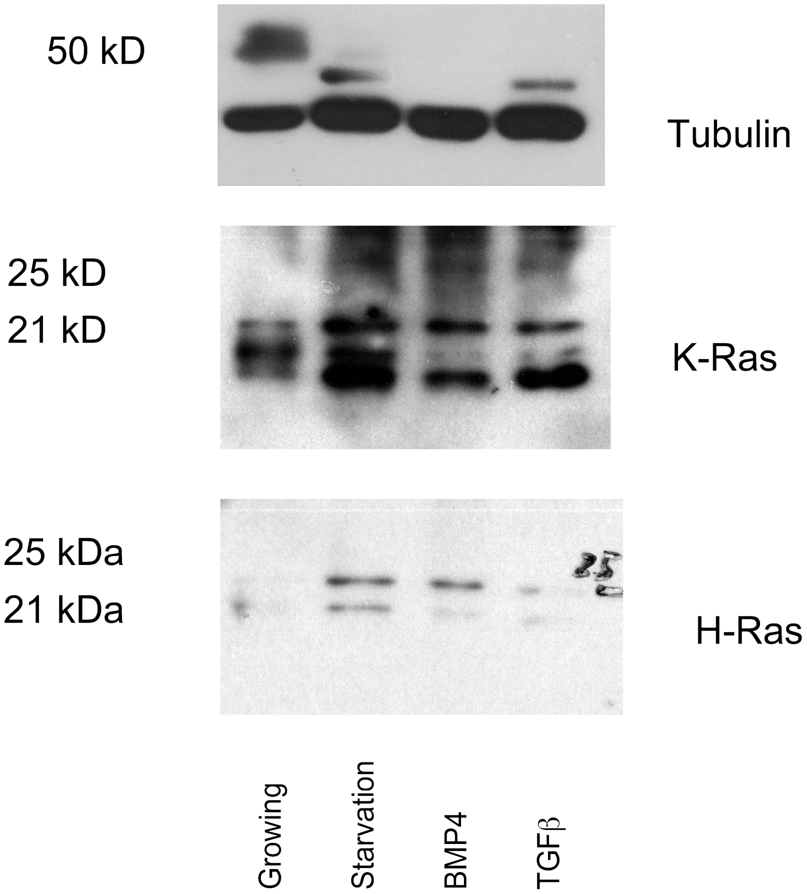

Figure S5. TGFβ treatment induces H-Ras and K-Ras in cultured astrocytes. To examine the effect of cytokines as alternative sources of ROS on astrocytes responses, cultured astrocytes were treated with 0.1–10 ng/mL TGFβ and BMP4 or serum deprivation and Western blot analysis of H- and K-Ras was performed. Primary astrocytes were cultured as described and subsequently serum starved and/or exposed to BMP4 and TGFβ for 20 min in serum-deprived medium. RIPA extracts were analyzed by immunoblot with specific antibodies to H-Ras and K-Ras, tubulin as loading control.

antioxidants

MDPI

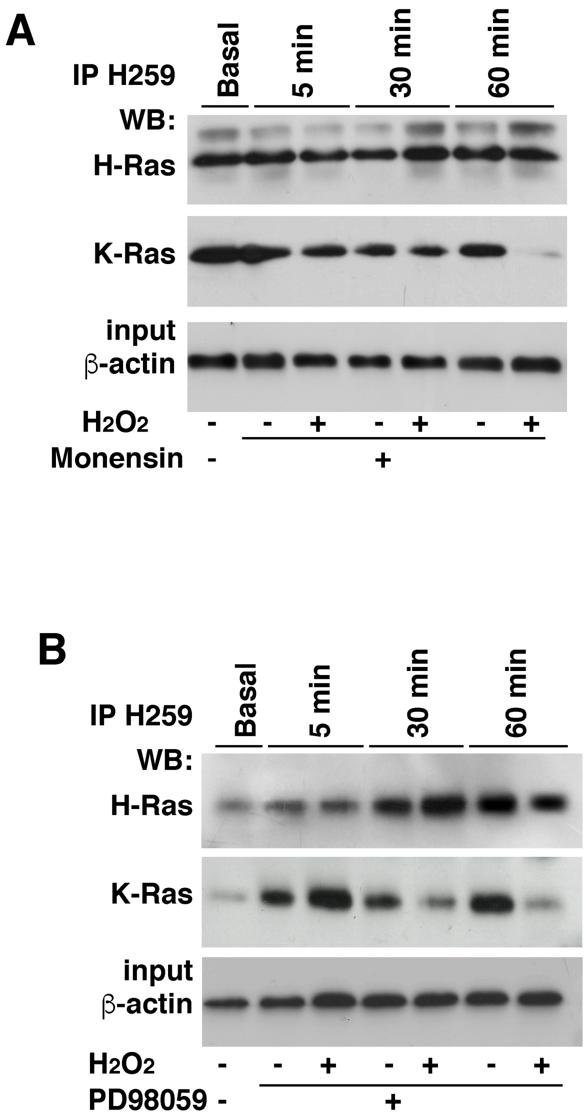

Figure S6. H- and K-Ras protein levels in cells exposed to H_2O_2 and treated with Monensin and MEK inhibitor, PD98059. (**A**). Primary astrocytes were exposed 5, 30 and 60 min to 500 μM H_2O_2, in presence or absence of Monensin. Cytosolic fraction were immunoprecipitated with H259 antibody and analyzed by immunoblot with specific antibodies to H-Ras and K-Ras. Western blot analysis of monoclonal β-actin was performed in the same experiment and used as loading control. (**B**). Primary astrocytes were exposed for 5, 30 and 60 min to 500 μM H_2O_2, in presence or absence of MEK inhibitor, PD98059. Total extracts were immunoprecipitated with H259 antibody and analyzed by immunoblot with specific antibodies to H-Ras and K-Ras. Western blot analysis of monoclonal β-actin was performed in the same experiment and used as loading control.
